# Polygenic risk for schizophrenia and subjective well-being in a general population sample

**DOI:** 10.1017/S0033291725000911

**Published:** 2025-05-02

**Authors:** Oona Serimaa, Liisa Keltikangas-Järvinen, Leo-Pekka Lyytikäinen, Jarmo Hietala, Elina Sormunen, Mika Kähönen, Olli Raitakari, Terho Lehtimäki, Aino Saarinen

**Affiliations:** 1 Department of Psychology and Logopedics, Faculty of Medicine, University of Helsinki, Finland; 2 Department of Clinical Chemistry, Fimlab Laboratories, and Finnish Cardiovascular Research Center, Tampere, Finland; 3 Department of Cardiology, Heart Center, Tampere University Hospital, Tampere, Finland; 4 Faculty of Medicine and Health Technology, Tampere University, Tampere, Finland; 5 Department of Psychiatry, University of Turku and Turku University Hospital, Turku, Finland; 6 Department of Clinical Physiology, Tampere University Hospital and Faculty of Medicine and Health Technology, Tampere University, Tampere, Finland; 7 Research Centre of Applied and Preventive Cardiovascular Medicine, University of Turku, Turku, Finland; 8 Department of Medicine, University of Turku and Division of Medicine, Turku University Hospital, Turku, Finland; 9 Centre for Population Health Research, University of Turku and Turku University Hospital, Turku, Finland

**Keywords:** life satisfaction, optimism, PRS, psychotic disorders, psychosis risk, pelf-acceptance

## Abstract

**Background:**

Previous evidence has reported associations of a polygenic risk score for schizophrenia (PRS_SCZ_) with negative developmental outcomes, such as psychiatric symptoms, adverse health behaviors, and reduced everyday functioning. We now investigated the relationship of PRS_SCZ_ with subjectively experienced well-being.

**Methods:**

Participants (n = 1866) came from the prospective population-based Young Finns Study (YFS). Subjective well-being in adulthood was assessed in terms of life satisfaction, optimism, and self-acceptance (when participants were 20–50 years old). A PRS_SCZ_ was calculated based on the most recent genome-wide association study on schizophrenia. Covariates included age, sex, early family environment, adulthood socioeconomic factors, and adulthood health behaviors.

**Results:**

The PRS_SCZ_ did not predict any domain of subjective well-being, including life satisfaction, optimism, and self-acceptance. After adding covariates in a stepwise manner or including/excluding participants with diagnosed non-affective psychotic disorders, all the associations remained non-significant. Age- and sex-interaction analyses showed that PRS_SCZ_ was not associated with subjective well-being in either sex or in any age between 20 and 50 years.

**Conclusions:**

While high PRS_SCZ_ has been linked to multiple adversities in previous studies, we did not find any association between high PRS_SCZ_ and subjective measures of life satisfaction, optimism, and self-acceptance.

## Introduction

The risk of developing schizophrenia is strongly linked to genetics, with heritability estimated at ~80% based on twin studies (Hilker et al., 2018). Candidate genes associated with schizophrenia have been identified (Farrell et al., 2015); however, results have not been documented in meta-analyses (Johnson et al., 2017). More recently, the genetics of schizophrenia have been studied using genome-wide association studies (GWAS) that analyze millions of common genetic variations or single nucleotide polymorphisms (SNPs) to establish their association with schizophrenia (Legge et al., 2021). Based on the GWAS studies, it is possible to calculate polygenic risk scores for schizophrenia (PRS_SCZ_). A significant breakthrough identified 83 new loci and 128 SNP differences linked to schizophrenia (Consortium, 2014). Further, the most extensive GWAS study on schizophrenia identified 287 loci, focusing on genes like GRIN2A, SP4, STAG1, and FAM120A, which are expressed in central nervous system neurons and act as excitatory or inhibitory factors, playing a pivotal role in neuronal functions (Trubetskoy et al., 2022).

GWAS studies on schizophrenia have shown that PRS_SCZ_ can explain between 7.7% and 33% of the variance in liability to schizophrenia (Lee et al., 2012; Legge et al., 2021; Purcell et al., 2009). Among those who have developed the illness, a high PRS_SCZ_ is associated with more severe symptoms (Fanous et al., 2012; Jonas et al., 2019) and a more severe disease course (Jonas et al., 2019; Meier et al., 2016). Research also indicates that high levels of PRS_SCZ_ increase susceptibility to other mental health conditions, including depression, anxiety, panic symptoms, and bipolar disorder, either in childhood or adulthood (Crouse et al., 2021; Richards et al., 2019; Zheutlin et al., 2019). In terms of lifestyle adversities, high PRS_SCZ_ has been associated with a higher risk of smoking (Wang et al., 2020), alcohol consumption (Zheutlin et al., 2019), and sleep disturbances (Reed et al., 2019).

While PRS_SCZ_ is known to predict multiple adverse outcomes, research on its association with subjective well-being has remained scarce. There are, however, two studies examining the association of PRS_SCZ_ with positively toned traits such as creativity and social support. The studies have not only reported a correlation between high PRS_SCZ_ and greater creativity (Power et al., 2015) but also an association between high PRS_SCZ_ and lower perceived social support in middle adulthood (Saarinen et al., 2023).

There are many reasons why it is essential to study the relationship between PRS_SCZ_ and subjective well-being. First, an increasing proportion of the population has been purchasing genetic testing to be aware of their genetic risks for disorders. In 2022, a total of 197,779 genetic tests were made available globally (Halbisen & Lu, 2023). Furthermore, stigmatization is known to be strong in the context of psychotic disorders (Gronholm, Thornicroft, Laurens, & Evans-Lacko, 2017), even among those at risk for psychosis but without the disorder (Colizzi, Ruggeri, & Lasalvia, 2020). If genetic testing indicated an increased genetic risk for schizophrenia, some individuals might disclose this information to their employer, even in the absence of clinical symptoms (Lawrence et al., 2016). To prevent further stigmatization and negative stereotypical expectations among those aware of their genetic risk for psychoses, it is important to examine also potential positively toned outcomes of polygenic risk scores. Second, a large portion of research on PRS_SCZ_ focuses on register-based data related to socioeconomic outcomes (e.g. unemployment status) or healthcare visits – that is, register-based measures in which individuals with high PRS_SCZ_ are not directly heard. In particular, it is not known how they subjectively experience their life satisfaction. This is especially relevant, as people often seek medical care only when they perceive a decline in their quality of life rather than when the first symptoms appear (Gartland, Long, & Skevington, 2019). Thus, subjectively experienced quality of life plays a crucial role in help-seeking behavior.

In general, well-being measures can be roughly divided into two dimensions: affective well-being (the frequency of positive vs. negative effects) and cognitive well-being (cognitive evaluations of one’s life) (Diener, Suh, Lucas, & Smith, 1999). In our study, optimism referred to affective well-being and life satisfaction and self-acceptance to cognitive well-being. Life satisfaction is one of the strongest single indicators of well-being (Linley et al., 2009), reflecting the relationship between one’s life expectations and the life that has been realized (Pavot 1993). Optimism refers to a disposition toward positive future expectations and is associated with better recovery from stressful life situations (Carbone & Echols, 2017) and greater resilience in adverse circumstances (Gallagher, Long, & Phillips, 2020). Finally, self-acceptance refers to a tendency to acknowledge one’s own qualities without the need for change or self-blame and predicts one’s ability to let go of things that have been lost (Prigerson & Maciejewski, 2008).

We investigated whether PRS_SCZ_ predicts subjective well-being in terms of life satisfaction, optimism, and self-acceptance. We used the prospective, population-based sample of the Young Finns Study (YFS). Moreover, because the onset of a psychotic illness is known to cause a decrease in well-being (Fervaha et al., 2016), we also examined whether PRS_SCZ_ is related to subjective well-being among those not diagnosed with non-affective psychotic disorders. Since research on PRS_SCZ_ and well-being is scarce (only two studies resulted in contradictory findings), we did not formulate a specific research hypothesis.

## Methods

### Participants

The Young Finns Study (YFS) is an ongoing prospective study that started in 1980 (baseline assessment). Follow-ups have been conducted in 1983, 1986, 1989, 1992, 1997, 2001, 2007, 2011/2012, and 2018–2020. Originally, a total of 4320 participants were invited (born in 1962, 1965, 1968, 1971, 1974, or 1977), and 3596 of them participated in the baseline study. The sample was designed to include a population-based sample of non-institutionalized Finnish children, representative of the most crucial sociodemographic factors. In practice, the sampling was conducted in collaboration of five Finnish universities with medical schools (i.e. Universities of Helsinki, Turku, Tampere, Oulu, and Kuopio). The design and methods of the YFS study are described in more detail elsewhere (Raitakari et al., 2008).

The Declaration of Helsinki has been followed throughout the study. The study design has been approved by the ethical committees of all the Finnish universities conducting the study. All the participants or their parents (participants aged <18 years) provided informed consent before participation.

From the full sample of 3596 participants, we included all the participants who had data available on variables under investigation: that is, participants who had been genotyped in 2011 for calculating their polygenic risk for schizophrenia and who had data available on subjective well-being measures in at least one measurement point: optimism in 2011, life satisfaction in 2007 and/or 2011, and self-acceptance in 1997, 2011, and/or 2012. Additionally, the analyses were adjusted in a stepwise manner for covariates: age, sex, quality of early family environment (1980 and/or 1983), adulthood socioeconomic factors (2011), and adulthood health behaviors (2001, 2007, and/or 2011). This resulted in a stepwise decrease in the sample size when adding covariates in each model. Thus, the final sample size ranged from 878 to 1866 participants in the main analyses.

### Measures

#### Polygenic risk score for schizophrenia (PRS_SCZ_)

Polygenic risk for schizophrenia was calculated based on the most recent GWAS study on schizophrenia (Trubetskoy et al., 2022). In the calculation, we used the PRS-CS method (Ge et al., 2019), which infers posterior SNP effect sizes under continuous shrinkage (CS) priors using GWAS summary statistics and an external LD reference panel. The results of the late available GWAS on schizophrenia (Trubetskoy et al., 2022) were used as SNP summary statistics, and HapMap 3 EUR was used as an external LD reference (Altshuler et al., 2010).

#### Subjective well-being

**Life satisfaction** was measured on a scale adapted from the Operation Family Study Questionnaire (Makkonen et al., 1981) in 2007 and 2011 (participants were 30–49 years old). The scale has been used also previously (Hintsa et al., 2006; Keltikangas-Järvinen & Heinonen, 2003; Makkonen et al., 1981). The self-assessment questionnaire contains three items measuring life satisfaction in three roles: as a spouse, employee, and parent (e.g. ‘Rate yourself as a parent’). Responses were given on a 5-point Likert scale (1 = very dissatisfied, 5 = very satisfied). In addition, the response scale included a response alternative of 0 (‘Does not apply to the respondent’) if, for example, the participant had no children or was not in working life. These values were coded as missing values. We calculated an average score (i.e. a life satisfaction index) over the three items for all the participants who had responded to at least one of the items in both follow-ups. Thus, higher values of the life satisfaction index indicated higher life satisfaction. Life satisfaction scores seemed to be moderately stable over time (r = 0.46 between the measurement years).

**Optimism** was measured in 2001 (when the participants were 24–39 years old) with the Revised Life Orientation Test-Revised (LOT) questionnaire (Scheier, Carver, & Bridges, 1994). The questionnaire contains six self-rated items (e.g. ‘I always have an optimistic attitude to the future’), including three reversed items (e.g. ‘If something can go wrong with me, it certainly will’). The items were answered on a 5-point Likert scale (0 = strongly disagree, 4 = strongly agree). In this study, an average score was calculated over the items for all the participants who had responded to at least half of the items. The LOT questionnaire has shown adequate validity and reliability (Scheier et al., 1994). In our dataset, its internal reliability was good (Cronbach’s α = 0.78) in accordance with previous studies (Heinonen, Räikkönen, & Keltikangas-Järvinen, 2005; Serlachius et al., 2017).

**Self-acceptance** was measured with the Self-Acceptance versus Self-Striving Scale which is a subscale of the Temperament and Character Inventory (TCI) (Cloninger 1994). The scale was conducted in the follow-ups of 1997, 2001, and 2012 (when participants were aged 20–50 years). The scale contains 11 statements that were responded to a 5-point Likert scale (1 = strongly disagree, 5 = strongly agree). The items included also reversed statements (e.g. ‘I wish I was smarter than anyone else’) which were reversely scored. Thus, higher scores on the scale referred to higher self-acceptance. In this study, the scale had high internal reliability (Cronbach’s α = 0.82–0.83 in different measurement years). The scores of self-acceptance were relatively stable over the 15-year follow-up (r = 0.59–0.67 between the measurement years).

#### Psychiatric diagnoses

In our supplementary analyses, we excluded participants who had been diagnosed with a non-affective psychotic disorder. Participants’ psychiatric diagnoses that had required hospital care were collected up to 2017 from the Care Register for Health Care (also known as the Finnish Hospital Discharge Register). In 2017, the participants were 40–55 years old and, thus, older than the typical onset age of schizophrenia (Ochoa et al., 2012). In the register, diagnoses were given by the prevailing diagnostic classification (ICD-8, ICD-9, or ICD-10). ICD diagnoses were converted to DSM-IV diagnoses, and this conversion is described in more detail elsewhere (Sormunen et al., 2017). Diagnoses were grouped into the following categories: (1) non-affective psychotic disorders, (2) substance-related disorders, (3) affective disorders (mood and anxiety disorders), and (4) personality disorders. Participants with several psychiatric diagnoses were categorized into only one of the categories in the following priority order: non-affective psychoses (DSM-IV 295, 297, 298), personality disorders (DSM-IV 301), affective disorders (mood and anxiety disorders, DSM-IV 296, 300, 311), and substance-related disorders (DSM-IV 291, 303, 292, 304, 305). The register is shown to cover most psychiatric diagnoses (Sund, 2012) and has been used previously for research purposes (Suvisaari, Haukka, Tanskanen, & Lönnqvist, 1999). In this study, we used a dichotomous variable of non-affective psychoses (0 = no, 1 = yes).

#### Covariates

**The quality of the early family environment** was assessed using three cumulative risk scores for (1) stressful life events, (2) adverse socioeconomic circumstances, and (3) unfavorable emotional atmosphere. Information on the early family environment was prospectively assessed in 1980/1983 with questionnaires presented to the parents. The cumulative risk score for *stressful life events* included changing residence, parental divorce, parental death, parental hospitalization in the past 12 months, and the child’s hospitalization due to illness or injury. The cumulative score for *adverse socioeconomic circumstances* included parents’ low occupational status, low educational, unstable employment situation, and overcrowded living conditions. The cumulative score for *unfavorable emotional atmosphere* included emotional distance between the child and parent, parental tolerance toward the child, strict disciplinary actions toward the child, parental life dissatisfaction, parents’ mental disorder, and parents’ frequent alcohol intoxication. When calculating the cumulative risk scores, each single variable (e.g. family income) was first standardized within each age cohort (mean = 0, SD = 1), and then an average score of these standardized variables was calculated. Thus, regardless of the original measurement scale, each factor was equally weighted in the cumulative risk scores (e.g. emotional distance between child and parent and parental life dissatisfaction were similarly weighted). The cumulative risk scores have been used also previously and reported there with further details (Saarinen et al., 2022).

**Adulthood socioeconomic factors** (2011) included annual income and educational level. Annual income was assessed with a 13-point scale (1 = < 5000 €, 13 = >60 000 €). Educational level included three classes (1 = comprehensive school including the first nine school years; 2 = occupational school or high school; 3 = academic level such as university or college). Each socioeconomic variable was added as a separate time-invariant covariate to the analyses.

**Adulthood health behaviors** included alcohol consumption, physical activity, and smoking that were assessed in 2001, 2007, and 2011. *Alcohol consumption* was assessed with self-report questions on consumption of 1/3 cans or bottles of beer, glasses (12 cl) of wine, and 4 cl shots of liquor or strong alcohol during the past week. The total alcohol consumption index has been used also previously and described in detail elsewhere (Juonala et al., 2009). *Physical activity* was assessed by inquiring about participants’ frequency and intensity of leisure-time physical activity and participation in organized sports exercises. The index of physical activity has been described elsewhere (Rovio et al., 2018). *Smoking* was assessed with a 5-point Likert scale ranging from 1 = (daily smoking) to 5 (never smoked). We formed a dichotomous variable (1 = daily smoking, 0 = not daily smoking). For each domain of health behaviors (alcohol consumption, physical activity, and smoking), an average score over the follow-ups was calculated for all participants who had data available in at least one measurement year.

### Statistical analyses

Statistical analyses were conducted using SPSS (version 29). First, pairwise correlations between the study variables were examined using Pearson/Spearman correlation coefficients. In addition, attrition over the prospective follow-up was analyzed using independent samples t-tests and χ^2^ tests. In attrition analyses, ‘included participants’ consisted of the participants who were included in at least one statistical analysis, while ‘non-participants’ referred to those who were excluded from all the analyses due to missing values in the study variables.

Next, linear regression analyses were used to investigate whether PRS_SCZ_ predicts life satisfaction or optimism. A total of three models were made: Models 1 were adjusted for sex and age, Models 2 also for the cumulative risk scores of early family environment, and Models 3 also for adulthood socioeconomic factors (annual income, occupational level, educational level) and health behaviors (alcohol consumption, physical activity, and smoking status). These covariates were selected because it is known that early family environment (Flèche, Lekfuangfu, & Clark, 2021), socioeconomic factors (Pinquart & Sörensen, 2000), and health behaviors (Grant, Wardle, & Steptoe, 2009) are associated with well-being and may act as potential confounders. The assumptions of linear regression analyses were fulfilled: the residuals followed an approximately normal distribution and the variances of the residuals were approximately homogeneous. We also calculated values of R squared change for PRS_SCZ_, indicating the percentage of the variation in subjective well-being that was explained by PRS_SCZ_.

Finally, we predicted the trajectory of self-acceptance by PRS_SCZ_ using growth curve models. The models contain both fixed and random effects. Fixed effects can be interpreted similarly to regression coefficients and, in this study, were estimated for PRS_SCZ_ and all the covariates. In addition, age^2^ was included as a fixed effect since self-acceptance was assumed to possibly have curvilinear changes over age. Random effects included variance of repeated measures and variance of intercept (between-individual variance in the constant term). The structure of the random effects covariance matrix was defined to be scaled identity. Regarding control variables, we added covariates in a stepwise manner The results were illustrated with graphs drawn with Stata SE version 18.

## Results

### Sample statistics

The descriptive statistics of the study variables are shown in Table 1. Participants were on average 41.3 years old (in 2011), and 52.1% of them were biologically male. Most of the participants had a second-level education (42.0%). Approximately 1.2% of the participants had been diagnosed with a non-affective psychotic disorder. On average, the participants seemed to be rather satisfied with their lives (mean = 3.96). In addition, self-acceptance seemed to increase over age. The pairwise correlations between the study variables can be found in Supplementary Table 1.

**Table 1.** Descriptive statistics of the study variables

	Frequency (%)	Mean (SD)	Measurement range
Sex (Female)	967 (47.9)		
Age (2011)		41.31 (5.05)	34–49
Polygenic risk for schizophrenia		−0.02 (1.00)	−4.72–2.94
Optimism		2.85 (0.67)	0–5
Life-satisfaction		3.96 (0.72)	1–5
Self-acceptance			
1997		3.63 (0.71)	1–5
2001		3.83 (0.67)	1–5
2011		3.89 (0.60)	1–5
Educational level			
Comprehensive school	276 (14.9)		
Occupational school or high school	779 (42.0)		
Academic level	505 (27.2)		
Level of income		7.53 (3.19)	1–13
Cumulative risk scores of early family environment			
Stressful life events		0.00 (0.41)	−0.50–2.24
Unfavorable emotional family atmosphere		0.00 (0.53)	−1.26–2.59
Socioeconomic adversities		0.06 (0.63)	−1.35–2.59
Health behaviors			
Alcohol consumption		0.90 (1.20)	0–14.29
Physical activity		8.90 (1.67)	5–14.67
Smoking status	303 (16.3)		
Diagnosis of non-affective psychotic disorder	22 (1.2)		

Attrition analyses showed that included participants and non-participants (i.e. those excluded from analyses due to missing values) did not differ in terms of subjective well-being such as life satisfaction (p = 0.63), optimism (p = 0.53), or self-acceptance (p = 0.57–0.86). Also, there was no attrition bias in age (p = 0.11), sex (p = 0.14), health behaviors (p = 0.10–0.98), or cumulative risk scores for stressful life events (p = 0.68) or unfavorable emotional family atmosphere (p = 0.76). Participants had, however, slightly lower scores of PRS_SCZ_ (p < 0.05) and were less likely to have been diagnosed with a non-affective psychotic disorder (p < 0.001) than non-participants. In addition, participants had slightly higher socioeconomic conditions both in childhood (p < 0.001) and adulthood in terms of higher education (p < 0.001) and higher income (p < 0.05) when compared with non-participants.

### Main analyses

The results of the linear regression analyses when predicting life satisfaction are presented in Table 2. To summarize, PRS_SCZ_ did not predict life satisfaction in Model 1 (p = 0.15), Model 2 (p = 0.18), or Model 3 (p = 0.37). Table 3 shows the results of linear regression analyses when predicting optimism. Again, PRS_SCZ_ did not statistically significantly predict optimism in any of the three models (p = 0.82 in Model 1, p = 0.71 in Model 2, and p = 0.54 in Model 3).

**Table 2.** Results of linear regression analyses when life satisfaction was predicted by the polygenic score for schizophrenia (PRS_SCZ_)

	Model 1 (n = 1520)			Model 2 (n = 1462)			Model 3 (n = 992)		
	B	95% confidence interval	*p*	B	95% confidence interval	*p*	B	95% confidence interval	*p*
Intercept	3.81	3.54; 4.06	< 0.001	3.81	3.55; 4.07	< 0.001	3.41	2.97; 3.85	< 0.001
PRS_SCZ_	−0.02	−0.05; 0.01	0.15	−0.02	−0.05; 0.01	0.18	−0.02	−0.05; 0.02	0.37
Sex^a^	0.02	−0.04; 0.09	0.48	0.02	−0.04; 0.09	0.45	0.05	−0.04; 0.13	0.27
Age	0.00	−0.00; 0.01	0.22	0.00	−0.00; 0.01	0.25	0.00	−0.00; 0.01	0.28
Unfavorable emotional atmosphere^b^				−0.10	−0.16; −0.04	< 0.001	−0.08	−0.15; −0.01	0.03
Socioeconomic adversities^b^				−0.03	−0.08; 0.02	0.21	−0.02	−0.08; 0.05	0.59
Stressful life events^b^				−0.07	−0.15; 0.00	0.06	−0.06	−0.15; 0.04	0.23
Educational level							−0.04	−0.11; 0.04	0.36
Level of income							0.03	0.01; 0.04	< .001
Alcohol consumption							−0.03	−0.06; 0.01	0.20
Physical activity							0.03	0.01; 0.06	0.01
Smoking status							0.05	−0.08; 0.17	0.47

*Note:* B refers to an unstandardized coefficient.

a Female as the reference group.

b Cumulative risk scores of early family environment.

**Table 3.** Results of linear regression analyses when optimism was predicted by the polygenic score for schizophrenia (PRS_SCZ_)

	Model 1 (n = 1414)			Model 2 (n = 1365)			Model 3 (n = 886)		
	B	95% confidence interval	*p*	B	95% confidence interval	*p*	B	95% confidence interval	*p*
Intercept	2.69	2.40; 2.98	< 0.001	2.67	2.38; 2.97	< 0.001	1.55	1.08; 2.02	< 0.001
PRS_SCZ_	0.00	−0.03; 0.04	0.82	0.01	−0.03; 0.04	0.71	0.01	−0.03; 0.05	0.54
Sex^a^	0.00	−0.07; 0.07	0.92	−0.00	−0.07; 0.07	0.94	0.03	−0.06; 0.12	0.52
Age	0.00	−0.00; 0.01	0.28	0.00	−0.00; 0.01	0.26	0.01	−0.00; 0.02	0.02
Unfavorable emotional atmosphere^b^				−0.13	−0.19; −0.06	< 0.001	−0.06	−0.13; 0.02	0.14
Socioeconomic adversities^b^				−0.17	−0.23; −0.12	< 0.001	−0.09	−0.15; −0.02	0.01
Stressful life events^b^				−0.01	−0.10; 0.08	0.79	0.04	−0.06; 0.14	0.41
Educational level							0.11	0.03; 0.19	0.01
Level of income							0.03	0.02; 0.05	< 0.001
Alcohol consumption							−0.07	−0.11; −0.03	< 0.001
Physical activity							0.06	0.03; 0.08	< 0.001
Smoking status							0.03	−0.10; 0.16	0.64

*Note:* B refers to an unstandardized coefficient.

a Female as the reference group.

b Cumulative risk scores of early family environment.

As an additional analysis, we investigated whether sex or age could modify the associations of PRS_SCZ_ with life satisfaction or optimism. For this, the interaction term of PRS_SCZ_*sex or PRS_SCZ_*age was separately added to the model. None of the interactions were statistically significant (p = 0.12–0.80), indicating that the association of PRS_SCZ_ with life satisfaction and optimism was non-significant in both sexes and over the age range of our sample (30–49 years).

**Figure 1.**
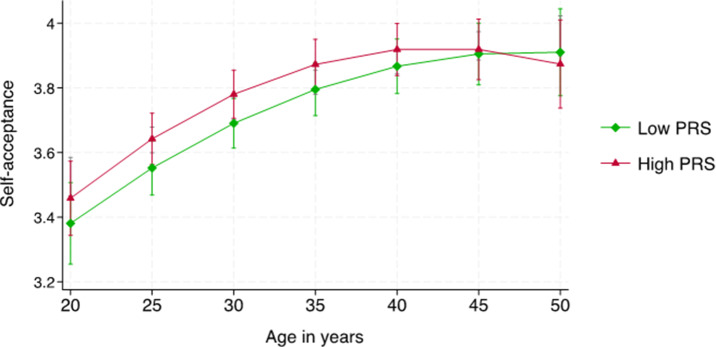
Estimated means of self-acceptance over age separately for individuals with low (−1 SD) or high (+ 1 SD) scores of polygenic risk for schizophrenia (PRS).

The results of the growth curve models are presented in Table 4. Age (p < 0.001) and age squared (p < 0.001) were significant predictors of self-acceptance, indicating that self-acceptance increased in a curvilinear way over age (p < 0.001). PRS_SCZ_ did not predict the trajectory of self-acceptance in any of the three models (p = 0.23 in Model 1, p = 0.40 in Model 2, and p = 0.60 in Model 3). As an additional analysis, we examined sex or age modified association of PRS_SCZ_ with self-acceptance. For this, the interaction term of PRS_SCZ_*sex or PRS_SCZ_*age^2^ was added to the model. None of the interactions were statistically significant (p = 0.61–0.99), indicating that the association of PRS_SCZ_ with a trajectory of self-acceptance was non-significant in both sexes and over the age range of our sample (20–50 years). The results of the growth curve models are illustrated in Figure 1.

**Table 4.** Results of growth curve models when predicting self-acceptance by the polygenic risk score for schizophrenia

		Model 1 (n = 1866)			Model 2 (n = 1794)			Model 3 (n = 1053)		
		B	95% confidence interval	*p*	B	95% confidence interval	*p*	B	95% confidence interval	*p*
Fixed effects										
	Intercept	2.74	2.51; 2.98	< 0.001	2.75	2.51; 2.99	< 0.001	2.62	2.23; 3.00	< 0.001
	PRS_SCZ_	0.02	−0.01; 0.05	0.23	0.01	−0.02; 0.04	0.40	0.01	−0.03; 0.05	0.60
	Sex^a^	−0.06	−0.11; −0.00	0.04	−0.05	−0.11; 0.00	0.06	−0.02	−0.10; 0.06	0.58
	Age	0.05	0.03; 0.06	< 0.001	0.05	0.03; 0.06	< 0.001	0.05	0.04; 0.07	< 0.001
	Age squared	0.00	−0.00; 0.00	< 0.001	0.00	−0.00; 0.00	< 0.001	−0.00	−0.00; 0.00	< 0.001
	Unfavorable emotional atmosphere^b^				−0.11	−0.16; −0.06	< 0.001	−0.07	−0.14; −0.00	0.05
	Socioeconomic adversities^b^				−0.04	−0.08; 0.01	0.09	−0.03	−0.09; 0.03	0.30
	Stressful life events^b^				−0.07	−0.14; −0.00	0.04	−0.05	−0.13; 0.04	0.31
	Educational level							0.04	−0.03; 0.12	0.23
	Level of income							−0.00	−0.01; 0.01	0.78
	Alcohol consumption							−0.03	−0.07; 0.00	0.10
	Physical activity							−0.00	−0.03; 0.02	0.73
	Smoking status							0.02	−0.09; 0.14	0.69
Random effects										
	Variance of intercept	0.28	0.26; 0.31	< 0.001	0.28	0.25; 0.30	< 0.001	0.26	0.24; 0.29	< 0.001
	Repeated measures variance	0.16	0.15; 0.17	< 0.001	0.16	0.15; 0.17	< 0.001	0.16	0.15; 0.17	< 0.001

*Note:* B refers to an unstandardized regression coefficient.

a Female as the reference group.

b Cumulative risk scores of early family environment.

### Sensitivity analyses

As additional analyses, we repeated the main analyses in a subsample of participants who had not been diagnosed with non-affective psychotic disorder. This was done since the onset of a psychotic illness is known to commonly cause a significant drop in subjective well-being (Fervaha et al., 2016). Thus, here we examined whether PRS_SCZ_ is associated with subjective well-being (life satisfaction, optimism, and self-acceptance) in individuals without a history of non-affective psychosis.

The results can be found in Supplementary Tables 2–4. To summarize, the main results were mostly replicated: in any model, PRS_SCZ_ did not predict optimism (p = 0.73 in Model 1, p = 0.61 in Model 2, and p = 0.49 in Model 3), life satisfaction (p = 0.15 in Model 1, p = 0.19 in Model 2, and p = 0.40 in Model 3), or self-acceptance (p = 0.28 in Model 1, p = 0.43 in Model 2, and p = 0.67 in Model 3).

Finally, we also reran the analyses so that all participants with psychiatric diagnoses required hospital care were excluded from the sample (i.e. non-affective psychoses, mood disorders, substance use disorders, personality disorders). The results were replicated: PRS was not associated with optimism (p = 0.628 in Model 1, p = 0.563 in Model 2, and p = 0.436 in Model 3), life satisfaction (p = 0.065 in Model 1, p = 0.090 in Model 2, and p = 0.252 in Model 3), or self-acceptance (p = 0.286 in Model 1, p = 0.464 in Model 2, and p = 0.672 in Model 3).

### Power estimation

Given the null results, it was necessary to estimate statistical power in our analyses. Therefore, we conducted post hoc power analyses to determine the minimum effect sizes detectable in our sample. Based on the sample sizes (n = 1502 for life satisfaction, n = 1414 for optimism, and n = 1866 for self-acceptance), a recommended power estimate of 80%, and a statistical significance threshold of p = 0.05, the minimum detectable squared partial correlations of PRS_SCZ_ were 0.0051, 0.0055, and 0.0042 for life satisfaction, optimism, and self-acceptance, respectively. These correspond to effect sizes of 0.0052, 0.0056, and 0.0042, respectively. Taken together, our sample size allowed us to detect associations between PRS_SCZ_ and well-being indicators with effect sizes as small as 0.0042–0.0052.

## Discussion

This study investigated, for the first time, the association between the polygenic risk for schizophrenia (PRS_SCZ_) and subjectively experienced well-being. Contrary to our hypotheses, PRS_SCZ_ did not predict aspects of subjective well-being such as life satisfaction, optimism, and self-acceptance. We found no associations between PRS_SCZ_ and well-being in any model with stepwise inclusion of covariates (age, sex, early family environment, adulthood socioeconomic factors, adulthood health behaviors), nor when including or excluding individuals who had developed non-affective psychotic disorders. Additionally, PRS_SCZ_ was not associated with subjective well-being in either sex or in any age between 20 and 50 years. In sum, our study showed that individuals with a higher PRS_SCZ_ reported being just as satisfied, optimistic, and self-accepting as those with a lower PRS_SCZ_.

While previous evidence has shown associations between PRS_SCZ_ and other mental disorders, including depression, anxiety, panic symptoms, bipolar disorder (Crouse et al., 2021; Richards et al., 2019; Zheutlin et al., 2019), as well as adverse health behaviors such as alcohol consumption (Zheutlin et al., 2019) and sleep disturbances (Reed et al., 2019), we found no association between PRS_SCZ_ and subjective well-being. Since it is well-known that experienced life satisfaction largely depends on the standards individuals set for their lives (Pavot 1993), individuals with a higher PRS_SCZ_ may possibly set more modest life expectations and find satisfaction from smaller and more attainable goals. Our results also align with previous reports that psychological ill-being and well-being constitute distinct dimensions (Ryff et al., 2006), indicating that the presence of mental symptoms may not necessarily reduce one’s sense of well-being.

Previous studies have reported a decrease in subjective well-being in individuals at familial risk for psychosis (Ellersgaard et al., 2020; Sin et al., 2016) and also after the onset of a psychotic disorder such as schizophrenia (Fervaha et al., 2016). We found that individuals at polygenic risk for schizophrenia do not experience lower levels of life satisfaction, optimism, or self-acceptance. This may have many explanations. First, a crucial factor contributing to the reduced well-being of psychotic patients seems to be stigmatization (Degnan, Berry, Humphrey, & Bucci, 2021) that, in turn, requires awareness of the disorder or an elevated risk for it. Unlike individuals with either familial risk or symptoms of psychosis, individuals with a high PRS_SCZ_ may not necessarily be aware of their risk for schizophrenia. Second, the offspring of psychotic patients are known to be exposed to illness-related adversities, such as parent relapses, difficulties in maintaining daily routines, or being emotionally present (da Silva et al., 2022; Strand, Boström, & Grip, 2020). Those with a high PRS_SCZ_, on the other hand, may not necessarily have close family members with psychotic disorders and may not encounter similar illness-related sources of stress.

A relevant question is whether our null results might result from limited statistical power. According to our power analyses, our sample size allowed us to detect associations between PRS_SCZ_ and well-being indicators with effect sizes as small as 0.0042–0.0052, corresponding to minimum detectable squared partial correlations of 0.0042–0.0055, respectively. If there had been an association with an effect size smaller than that, it would be reasonable to question whether such an association holds any practical significance. Additionally, our sample has enabled the detection of statistically significant associations with psychosocial outcomes in our previous studies: for example, individual differences in PRS_SCZ_ have explained variance in magical thinking (Saarinen et al., 2022), social support from close networks (Saarinen, Hietala, et al., 2023), and accelerated epigenetic aging in the interaction of social dispositions (Saarinen, Hietala, et al., 2023). Taken together, while the risk of type II error should be considered, we argue that our null results are unlikely to be solely due to a lack of statistical power.

Regarding limitations, there was participant drop-out over the follow-up from the baseline measurement in 1980, resulting in a sample size of 1866 participants in our study. Our attrition analyses, however, did not identify any systematic drop-out with respect to most of the study variables. That is, participants and non-participants (i.e. those excluded from the analyses due to missing values) did not differ in indicators of subjective well-being, age, sex, health behaviors, or most qualities of early family environment. However, included participants were less likely to have been diagnosed with a non-affective psychotic disorder, had slightly lower PRS_SCZ_ scores, and were living in slightly more advantaged socioeconomic conditions when compared with those who dropped out. Drop-out has been a well-documented issue in other prospective studies as well. When examining participant drop-out, previous reports on prospective datasets have generally found that it does not cause significant bias in results (de Graaf et al., 2000; Tambs et al., 2009). Researchers have concluded that ‘differential loss to follow-up rarely affects estimates of association’ (Saiepour et al., 2019).

The concept of subjective well-being encompasses many different definitions, making it challenging to establish a unified definition. First, some studies have included the absence of negative affect, such as sadness or envy, in their well-being measurements. However, we did not include assessments of negative affect, as they may confound with affective disorders that are known to correlate with PRS_SCZ_ (Richards et al., 2019). Second, while there have been attempts to assess subjective well-being through neural correlates, such as ‘brain fingerprints’ (Jung et al., 2022; Saarimäki et al., 2016), this method appears to require further development before it can produce reliable estimates of emotional well-being. Third, while some studies have evaluated well-being on the basis of external measures, such as wealth, recreational activities, or access to healthcare. A key strength of our study is that we were able to assess well-being based on individuals’ own subjective experiences over many follow-ups.

In a broader context, previous research has focused on the associations of PRS_SCZ_ with a range of negative developmental pathways, including mental disorders, lifestyle challenges, domains of reduced functioning, and an accumulation of other adversities. This study provides a novel positive perspective by demonstrating that individuals with high PRS_SCZ_ are, on average, as satisfied with their well-being as those with a lower PRS_SCZ_.

## Supporting information

Serimaa et al. supplementary material Serimaa et al. supplementary material

## Data Availability

The Cardiovascular Risk in Young Finns (YFS) dataset comprises health-related participant data, and their use is therefore restricted under the regulations on professional secrecy (Act on the Openness of Government Activities, 612/1999) and on sensitive personal data (Personal Data Act, 523/1999, implementing the EU data protection directive 95/46/EC). Due to these legal restrictions, the data from this study cannot be stored in public repositories or otherwise made publicly available. However, data access may be permitted on a case-by-case basis upon request. Data sharing outside the group is done in collaboration with the YFS group and requires a data-sharing agreement. Investigators can submit an expression of interest to the chairman of the publication committee (Prof. Mika Kähönen, Tampere University, Finland, mika.kahonen@tuni.fi).

## Supplementary material

The supplementary material for this article can be found at http://doi.org/10.1017/S0033291725000911.
