# Supplementary material for: Polygenic risk for schizophrenia and subjective well-being in a general population sample
Source: Psychol Med. 2025 May 2;55:e133. doi: 10.1017/S0033291725000911 (PMC12094622; doi:10.1017/S0033291725000911)
Supplement: Serimaa et al. supplementary material [file S0033291725000911sup001.docx]

**Supplementary Table 1.** Pairwise correlations between the study variables.

|  | **1.** | **2.** | **3.** | **4.** | **5.** | **6.** | **7.** | **8.** | **9.** | **10.** | **11.** | | | **12.** | | | **13.** | | | |  |
| --- | --- | --- | --- | --- | --- | --- | --- | --- | --- | --- | --- | --- | --- | --- | --- | --- | --- | --- | --- | --- | --- |
| **1. Sex^a^** |  |  |  |  |  |  |  |  |  |  |  | | |  | | |  | | | |  |
| **2. Age** | -0.03 |  |  |  |  |  |  |  |  |  |  | | |  | | |  | | | |  |
| **3. PRS_SCZ_** | 0.02 | -0.06 |  |  |  |  |  |  |  |  |  | | |  | | |  | | | |  |
| **4. Optimism** | -0.00 | 0.08* | 0.00 |  |  |  |  |  |  |  |  | | |  | | |  | | | |  |
| **5. Life satisfaction** | 0.01 | 0.09* | -0.04 | 0.26** |  |  |  |  |  |  |  | | |  | | |  | | | |  |
| **6. Self-acceptance (2001)** | 0.01 | 0.08* | 0.06 | 0.35** | 0.18** |  |  |  |  |  |  | | |  | | |  | | | |  |
| **7. Education level** | 0.06* | -0.14** | 0.03 | 0.16** | -0.02 | 0.04 |  |  |  |  |  | | |  | | |  | | | |  |
| **8. Level of income** | -0.33** | 0.05 | -0.06 | 0.21** | 0.05 | -0.01 | 0.29** |  |  |  |  | | |  | | |  | | | |  |
| **9. STRESSrisk** | -0.03 | -0.03 | -0.03 | 0.03 | -0.02 | 0.01 | -0.02 | 0.00 |  |  |  | | |  | | |  | | | |  |
| **10. EMOTrisk** | 0.05 | -0.07 | 0.01 | -0.09* | -0.05 | -0.01* | -0.10* | -0.03 | 0.16** |  |  | | |  | | |  | | | |  |
| **11. SESrisk** | 0.01 | 0.06 | 0.02 | -0.11** | -0.02 | -0.04 | -0.31** | -0.21** | 0.06 | -0.01 |  | | |  | | |  | | | |  |
| **12. Alcohol consumption** | -0.36** | 0.06 | 0.07 | -0.12** | 0.02 | 0.01 | -0.01 | 0.10* | 0.06 | 0.11** | -0.09* | | |  | | |  | | | |  |
| **13. Physical activity** | -0.01 | -0.09* | -0.05 | 0.18** | 0.07 | -0.02 | 0.13** | 0.16** | -0.07 | -0.10* | -0.16** | | | 0.03 | | |  | | | |  |
| **14. Smoking** | -0.09** | -0.02 | 0.02 | -0.09** | -0.05 | -0.07* | -0.34** | -0.08** | 0.03 | 0.12** | 0.15** | | | 0.12** | | | -0.21** | | | |  |
| ** p < .01, * p < .05  This table includes all participants who were included in at least one data analysis (n = 1855).  ^a^ Spearman's rank correlations were used for categorical variables.  PRS_SCZ_ = Polygenic risk score for schizophrenia  EMOTrisk = Cumulative risk score for unfavorable emotional family atmosphere  SESrisk = Cumulative risk score for socioeconomic adversities  STRESSrisk= Cumulative risk score for stressful life events | | | | | | | | | | | |  |  |  |  |  |  |  |  |  | |

**Supplementary Table 2.** Results of linear regression analyses when predicting life satisfaction by the polygenic risk score for schizophrenia (PRS_SCZ_). Note: Participants diagnosed with non-affective psychosis were excluded.

|  | **Model 1**  (n = 1505) | | |  | **Model 2**  (n = 1448) | | |  | **Model 3**  (n = 983) | | |
| --- | --- | --- | --- | --- | --- | --- | --- | --- | --- | --- | --- |
|  | **B** | **95 % confidence interval** | ***p*** |  | **B** | **95 % confidence interval** | ***p*** |  | **B** | **95 % confidence interval** | ***p*** |
| Intercept | 3.83 | 3.56; 4.09 | < 0.001 |  | 3.83 | 3.57; 4.10 | < 0.001 |  | 3.47 | 3.04; 3.91 | < 0.001 |
| PRS_SCZ_ | -0.02 | -0.05; 0.01 | 0.15 |  | -0.02 | -0.05; 0.01 | 0.19 |  | -0.02 | -0.05; 0.02 | 0.40 |
| Sex^1^ | 0.01 | -0.05; 0.08 | 0.65 |  | 0.02 | -0.05; 0.08 | 0.60 |  | 0.04 | -0.04; 0.13 | 0.33 |
| Age | 0.00 | -0.00; 0.01 | 0.27 |  | 0.00 | -0.00; 0.01 | 0.31 |  | 0.00 | -0.00; 0.01 | 0.36 |
| Unfavorable emotional atmosphere^2^ |  |  |  |  | -0.10 | -0.16; -0.04 | < 0.001 |  | -0.08 | -0.16; -0.01 | 0.02 |
| Socioeconomic adversities^2^ |  |  |  |  | -0.03 | -0.08; 0.02 | 0.25 |  | -0.02 | -0.08; 0.04 | 0.55 |
| Stressful life events^2^ |  |  |  |  | -0.06 | -0.14; 0.02 | 0.12 |  | -0.04 | -0.14; 0.05 | 0.40 |
| Educational level |  |  |  |  |  |  |  |  | -0.04 | -0.12; 0.04 | 0.29 |
| Level of income |  |  |  |  |  |  |  |  | 0.03 | 0.01; 0.04 | < 0.001 |
| Alcohol consumption |  |  |  |  |  |  |  |  | -0.02 | -0.06; 0.01 | 0.22 |
| Physical activity |  |  |  |  |  |  |  |  | 0.03 | 0.01; 0.05 | 0.02 |
| Smoking status |  |  |  |  |  |  |  |  | 0.06 | -0.07; 0.18 | 0.37 |
| B refers to an unstandardized regression coefficient. ^2^ Cumulative risk scores of early family environment.  ^1^ Female as the reference group. | | | | | | | | | | | |

**Supplementary Table 3.** Results of linear regression analyses when predicting optimism by the polygenic risk score for schizophrenia (PRS_SCZ_). Note: Participants diagnosed with non-affective psychosis were excluded.

|  | **Model 1**  (n = 1399) | | |  | **Model 2**  (n = 1351) | | |  | **Model 3**  (n = 878) | | |
| --- | --- | --- | --- | --- | --- | --- | --- | --- | --- | --- | --- |
|  | **B** | **95 % confidence interval** | ***p*** |  | **B** | **95 % confidence interval** | ***p*** |  | **B** | **95 % confidence interval** | ***p*** |
| Intercept | 2.71 | 2.41; 3.00 | < 0.001 |  | 2.70 | 2.40; 2.99 | < 0.001 |  | 1.60 | 1.13; 2.07 | < 0.001 |
| PRS_SCZ_ | 0.01 | -0.03; 0.04 | 0.73 |  | 0.01 | -0.03; 0.05 | 0.61 |  | 0.01 | -0.03; 0.05 | 0.49 |
| Sex^1^ | -0.01 | -0.08; 0.07 | 0.87 |  | -0.01 | -0.08; 0.06 | 0.72 |  | 0.02 | -0.07; 0.11 | 0.63 |
| Age | 0.00 | -0.00; 0.01 | 0.33 |  | 0.00 | -0.00; 0.01 | 0.32 |  | 0.01 | -0.00; 0.02 | 0.03 |
| Unfavorable emotional atmosphere^2^ |  |  |  |  | -0.13 | -0.19; -0.06 | < 0.001 |  | -0.06 | -0.14; 0.01 | 0.11 |
| Socioeconomic adversities^2^ |  |  |  |  | -0.17 | -0.23; -0.11 | < o.001 |  | -0.08 | -0.15; -0.02 | 0.01 |
| Stressful life events^2^ |  |  |  |  | 0.00 | -0.09; 0.09 | 0.97 |  | 0.06 | -0.04; 0.15 | 0.26 |
| Educational level |  |  |  |  |  |  |  |  | 0.11 | 0.04; 0.19 | 0.01 |
| Level of income |  |  |  |  |  |  |  |  | 0.03 | 0.02; 0.04 | < 0.001 |
| Alcohol consumption |  |  |  |  |  |  |  |  | -0.07 | -0.11; -0.02 | < 0.001 |
| Physical activity |  |  |  |  |  |  |  |  | 0.05 | 0.03; 0.08 | < 0.001 |
| Smoking status |  |  |  |  |  |  |  |  | 0.04 | -0.09; 0.17 | 0.54 |
| B refers to an unstandardized regression coefficient. ^2^ Cumulative risk scores of early family environment.  ^1^ Female as the reference group. | | | | | | | | | | | |

**Supplementary Table 4.** Results of growth curve models when predicting self-acceptance by the polygenic risk score for schizophrenia (PRS_SCZ_). Note: Participants diagnosed with non-affective psychosis were excluded.

|  | | | |  | | **Model 1**  (n = 1842) | | | |  | **Model 2**  (n = 1772) | | |  | **Model 3**  (n = 1042) | | |
| --- | --- | --- | --- | --- | --- | --- | --- | --- | --- | --- | --- | --- | --- | --- | --- | --- | --- |
|  | | | |  | | **B** | | **95 % confidence interval** | ***p*** |  | **B** | **95 % confidence interval** | ***p*** |  | **B** | **95 % confidence interval** | ***p*** |
| **Fixed effects** | | |  | | |  | |  |  |  |  |  |  |  |  |  |  |
|  | | Intercept | | | | 2.73 | | 2.49; 2.97 | < 0.001 |  | 2.74 | 2.50; 2.98 | < 0.001 |  | 2.62 | 2.23; 3.01 | < 0.001 |
|  | | PRS_SCZ_ | | | | 0.02 | | -0.01; 0.04 | 0.28 |  | 0.01 | -0.02; 0.04 | 0.43 |  | 0.01 | -0.03; 0.04 | 0.67 |
|  | | Sex^1^ | | | | -0.06 | | -0.12; -0.00 | 0.04 |  | -0.05 | -0.11;0.00 | 0.06 |  | -0.03 | -0.11; 0.05 | 0.53 |
|  | | Age | | | | 0.05 | | 0.04; 0.06 | < 0.001 |  | 0.05 | 0.04; 0.06 | < 0.001 |  | 0.05 | 0.04; 0.07 | < 0.001 |
|  | | Age squared | | | | 0.00 | | -0.00; 0.00 | < 0.001 |  | 0.00 | -0.00;0.00 | < 0.001 |  | -0.00 | -0.00;0.00 | < 0.001 |
|  | | Unfavorable emotional atmosphere^2^ | | | |  | |  |  |  | -0.10 | -0.16; -0.05 | < 0.001 |  | -0.07 | -0.14; -0.00 | 0.04 |
|  | | Socioeconomic adversities^2^ | | | |  | |  |  |  | -0.04 | -0.08; 0.01 | 0.08 |  | -0.03 | -0.09; 0.03 | 0.31 |
|  | | Stressful life events^2^ | | | |  | |  |  |  | -0.07 | -0.14; -0.00 | 0.04 |  | -0.05 | -0.14; 0.04 | 0.28 |
|  | | Educational level | | | |  | |  |  |  |  |  |  |  | 0.04 | -0.03; 0.12 | 0.23 |
|  | | Level of income | | | |  | |  |  |  |  |  |  |  | -0.00 | -0.02; 0.01 | 0.74 |
|  | | Alcohol consumption | | |  | | |  |  |  |  |  |  |  | -0.03 | -0.07; 0.00 | 0.12 |
|  | | Physical activity | | |  | | |  |  |  |  |  |  |  | -0.00 | -0.03; 0.02 | 0.79 |
|  | | Smoking status | | | |  | |  |  |  |  |  |  |  | 0.02 | -0.10; 0.14 | 0.71 |
| **Random effects** | | |  | |  | |  |  |  |  |  |  |  |  |  |  |  |
|  | | Variance of intercept | | | | | 0.28 | 0.26; 0.31 | < 0.001 |  | 0.28 | 0.25; 0.30 | < 0.001 |  | 0.26 | 0.24; 0.29 | < 0.001 |
|  | | Repeated measures variance | | | | | 0.16 | 0.15; 0.17 | < 0.001 |  | 0.16 | 0.15; 0.17 | < 0.001 |  | 0.16 | 0.15; 0.17 | < 0.001 |
| B refers to an unstandardized regression coefficient. ^2^ Cumulative risk scores of early family environment.  ^1^ Female as the reference group. | | | | | | | | | | | | | | | | | |
